# Supplementary material for: Assessing depressive symptoms among people living with HIV in Yangon city, Myanmar: Does being a member of self-help group matter?
Source: PLoS One. 2021 Mar 18;16(3):e0248807. doi: 10.1371/journal.pone.0248807 (PMC7971502; doi:10.1371/journal.pone.0248807)
Supplement: Attachment — Submitted filename: Responses to Reviewers_final.docx [file pone.0248807.s002.docx]

**Responses to reviewers**

Thank you very much for taking the time to review the manuscript. We responded to your valuable comments as below:

**Reviewer #1**

| **No** | **Reviewer’s comment** | **Authors’ response** |
| --- | --- | --- |
| 1 | In Page 4, you defined adults as aged 15 and above. Please justify if this definition is according to national law in Myanmar. | In Myanmar, different definitions of an adult exist. In Myanmar Penal Code, aged 15 and above is regarded as an adult and with the parent’s consent, a girl with the age of 15 can marry. However, Myanmar has already ratified Child Right Convention, and Ministry of Social Welfare defines the age of 18 and above as an adult. To avoid possible confusion, we rewrite the sentence without using the word “adults.”  We amended the sentence as follows:  “In 2018, HIV prevalence was estimated to be 0.8% among those who aged between 15 and 49. In total, 170,000 people received ART out of 240,000 PLHIV in Myanmar.” (line 29-31, page 4) |
| 2 | Study design: to consider using the term of “comparative cross-sectional design” | We added the term “comparative” to the design.  “A comparative cross-sectional study was conducted in Yangon, Myanmar in 2017.” (line 59, page 5) |
| 3 | Please explain whether the 19-item Medical Outcome Study Social Support Survey (MOS-SSS) is available in Burmese version. | The Burmese version of Medical Outcome Study Social Support Survey (MOS-SSS) was used in this study. This MOS-SSS Burmese version has already been applied to previous studies in refugee camps along Thai-Burma Border (e.g., Reference number 45). Burmese is the official language in Myanmar and the majority use this language. We inserted the following paragraph in the manuscript.  “The Cronbach’s alpha coefficients for subscales in the Burmese version were 0.87 on the emotional subscale, 0.85 on the tangible subscale, 0.87 on the affectionate subscale, and 0.82 on the positive social interaction subscale.” (line 125-127, page 8) |
| 4 | Explanation of the clinics or hospital where the study was conducted is needed – this can be done by adding a section on study setting. | We explained the ART clinic in this study as follows:  “Study setting  This study was conducted in the venues of ART clinics. A structured questionnaire was administered to all of the participants through face-to-face interviews in the Burmese language by trained interviewers in private rooms at the ART clinics. The ART clinics included in this study offered HIV testing and prevention awareness sessions. When people were diagnosed with HIV positive, the clinic provided ART and counseling. ART clinics provided meeting space for self-help group members to discuss their challenges. Self-help groups were independent of ART clinics that provided treatment for them.” (line 66-74, page 5-6) |
| 5 | A clear explanation on participants recruitment (how those participants were recruited) is required. | We added an extra explanation of how PLHIV were recruited at ART clinic.  “Second, PLHIV were recruited by a convenience sampling method when they visited the clinics during the data collection period. The clinic reception staff announced this study in the waiting area. Those who were interested in the participation received explanations from the staff on the purpose of this study. Only those who were willing to participate in this study were sent to the interview rooms.” (line 89-95, page 6-7) |
| 6 | In page 9, you mentioned: “of the members, 89 PLHIV (44.3%) and 86 PLHIV (32.7%) of the non-members had depressive symptoms.” This sentence is unclear and need to be re-write. | We amended the sentence to improve the clarity as follows:  “Depressive symptoms were found among 89 members (44.3%) and 86 non-members (32.7%).” (line 167-168, page 10) |
| 7 | In page 13, title of Table 3 should be corrected. | We corrected the title of Table 3 as per your comment.  Table 3: Factors associated with having depressive symptoms stratified by the membership status of a self-help group (N=464) (line 212-213, page 14) |
| 8 | In page 15, you mentioned: “Self-help group members were more likely to have depressive symptoms compared with the non-members”. Please explain implications of this findings in the discussion. | As a result of adding the “type of clinic” variable in our regression model in Table 2 (by reflecting other reviewers’ comments), self-help group membership status is no longer statistically significant. We updated the discussion for implication from the finding.  “Being self-help group members was not associated with having depressive symptoms. However, members who registered at NGO clinics had a lower risk of having depressive symptoms, compared to members who registered at NAP/CBO clinics. The NAP/CBO clinics had fewer human and material resources than NGO funded clinics. Some members who registered at NAP/CBO clinics volunteered in these clinics. Some members who registered at NAP/CBO clinics volunteered in these clinics, for example, for tracing the lost-to-follow-up of PLHIV. This might cause stress and burden on the members. Health professionals at NAP/CBO clinics may need an opportunity to learn from NGO clinics regarding how NGOs were supporting self-help groups.” (line 227-235, page 16) |
| 9 | In page 16, you mentioned: “Among both members and non-members, women were more likely than men to have depression”. Further explanation on this finding is required. | Male-dominated social norm has prevailed in Myanmar at the household, community, and even state level. LGBT often face an issue of acceptance in the society. Consequently, people tend to blame women and LGBT, rather than men, among those infected with HIV.  “Under persistent social norm related to gender, once women and LGBT are infected with HIV, they may perceive a higher level of stress than men. To address their stress, health professional needs to take proactive roles in raising gender equality awareness in the community and encouraging family members of PLHIV to respect gender diversity.” (line 249-253, page 17) |
| 10 | There should be a discussion on implications of the study findings. | We modified the entire Discussion and Conclusion sections to add implications from the study findings. Point-to-point modifications were described as the responses to other comments in this file. |
| 11 | In page 18, you mentioned “PLHIV should check their mental health status during their visit to ART clinics”. Please explain how this can be possible. Please also include a description on the availability of the assessment for PLHIV in your study setting. | We added a new paragraph for a possible solution to improve PLHIV’s mental health status under scarcity in the professional resources.  “A self-help group is a prominent approach for mental health and substance use problems. While a self-help group and its peer-to-peer counselling are not an inferior alternative to professional mental health support, it is beneficial for the participants to receive professional training from experts in mental health. They can learn how to deal with depressive symptoms and internalized stigma as well as expand social support. In Myanmar, mental health professionals are available only in the central and some limited community levels, and their number is scarce. Therefore, it would be feasible that PLHIV in self-help groups and staff in the ART clinics receive training, rather than they find professionals who can be dispatched to the clinics.” (line 276-284, page 18) |

**Reviewer #2**

| **No** | **Reviewer’s comment** | **Authors’ response** |
| --- | --- | --- |
| 1 | The self-help group and non self-help group are not comparable, so it is looks like the author could not compare the risk factors of depression in the two groups. This is a big problem in study design. | We recruited PLHIV from three ART clinics regardless of their participation in a self-help group. Then, we asked if PLHIV joined a self-help group or not by a questionnaire survey. In this sense, we believe that we can investigate if participation in a self-help group was associated with depressive symptoms.  Moreover, we examined factors associated depressive symptoms using a pooled analysis that included members and non-members in the same dataset, in addition to the stratified models by the membership status. These models were used to confirm if membership status might affect the association between other covariates and the outcome. Since the background characteristics of members and non-members were different, these characteristics were controlled in multiple regression models. As this is the cross-sectional study, we did not address a causal relationship, and we mentioned it in the limitation section.  We amended the title to the following: “Assessing depressive symptoms among people living with HIV in Yangon, Myanmar: Does being a member of self-help group matter?” |
| 2 | Social support is a part of self help. Why is social support lower in self help group? Does it mean that self-help has no effect? | We used Medical Outcome Study Social Support Survey (MOS-SSS) scale to measure the level of social support. Some items in MOS-SSS, particularly emotional-informational support, could be improved through the participation in a self-help group. However, in this study, the level of social support was lower among self-help group members. PLHIV who have higher social support might not join self-help group as they might receive social support from family members or friends or colleagues. Those who needed social support might join to seek social support. This study used the cross-sectional design and it did not establish a causal relationship. We recommended a longitudinal study to examine the effect of self-help groups and social support among self-help group members for future.  We explained the source of social support in the limitation section as follows.  “This study measured the different types of social support using the validated scale. However, social support could not be interpreted as a cause of depressive symptom under the study design of this study. Investigate reasons behind the lower level of social support among self-help group members was beyond our scope. Future studies should be designed to examine the role of social support in the causal relationship between the participation in self-help groups and depressive symptoms.” (line 297-303, page 19) |
| 3 | In table 3, N=xxx ? | We corrected the title of Table 3.  “Table 3: Factors associated with having depressive symptoms stratified by the membership status of a self-help group (N=464)” (line 212-213, page 14) |

**Reviewer #3**

| **No** | **Reviewer’s comment** | **Authors’ response** |
| --- | --- | --- |
| 1 | Methods Provide context-specific information regarding the organization of self-help group in the selected ART clinics. Is there any difference in the way the self-help group is organized in the selected ART clinics that could affect the results of this study? | We added context-specific information regarding the organization of self-help groups in the newly added section of “Study setting.”  “This study was conducted in the venues of ART clinics. A structured questionnaire was administered to all of the participants through face-to-face interviews in Burmese language by trained interviewers in private rooms at the ART clinics. The ART clinics included in this study offered HIV testing and prevention awareness sessions. When people were diagnosed with HIV positive, the clinic provided ART and counseling. ART clinics provided meeting space for self-help group members to discuss their challenges. Self-help groups were independent of ART clinics that provided treatment for them.” (line 66-74, page 5-6) |
| 2 | Please include how many patients were receiving services from each of the selected ART clinics. | To protect confidentiality of their clients, ART clinics did not provide detailed information about patients. We could not include information about the number of patients at the selected ART clinics. |
| 3 | Provide the criteria that guided the purposive selection of the three selected ART clinics | We added explanations according to your comment. Assistant Director from National AIDS Programme (NAP) at the central level recommended three ART clinics that cooperated well with NAP and sent a regular report to NAP at both state and central levels.  “First, three ART clinics were purposively selected through consultation with the National AIDS Programme (NAP). Assistant Director from NAP recommended three ART clinics that cooperated well with NAP and sent a regular report to NAP.” (line 86-89, page 6)  This purposive selection was indicated as a limitation as follows:  “Second, PLHIV were purposively selected by staff at the ART clinics, without involvement by the authors. That is, data on the number of recruited and refused to participate in the study were not available. Also, the number of PLHIV participated at the ART clinics did not reflect the sizes of the clinics. The majority of PLHIV recruited in this study was from NGO type clinics. These may affect the representativeness of this study.” (line 289-294, page 18) |
| 4 | Was refusal to participation or non-response documented for this study? | Due to the procedure of participant recruitment, we could not obtain the information about any refusal. The interviewers waited at the interview room, and the receptionist from the ART clinics sent those who agreed to participate in the interview. We modified explanations about recruitment as follows:  “Second, PLHIV were recruited by a convenience sampling method when they visited the clinics during the data collection period. The clinic reception staff announced this study in the waiting area. Those who were interested in the participation received explanations from the staff on the purpose of this study. Only those who were willing to participate in this study were sent to the interview rooms.” (line 89-95, page 6-7)  We also explain this as a limitation.  “Second, PLHIV were purposively selected by staff at the ART clinics, without involvement by the authors. That is, data were not available on the number of recruited and refused to participate in the study. Also, the number of PLHIV participated at the ART clinics did not reflect the sizes of the clinics. The majority of PLHIV recruited in this study was from NGO type clinics. These may affect the representativeness of this study.” (line 289-294, page 18) |
| 5 | Analysis  The multiple logistic regression should include the variable “type of clinics” to control for its potential effect. Given that the majority of patients (82.5%) were recruited from a single ART clinic (NGO), I suggest that this variable be dichotomized into: NGO and Other clinics (NAP; CBO; Other). | We added the variable “type of clinic” in our analysis. We dichotomized this variable as suggested.  “In addition, the variable “type of clinic” was recorded as the organization of the ART clinic where PLHIV was recruited. It was used as dichotomized: NGO clinic and other (clinic run by NAP or a community-based organization).” (line 129-131, page 8) |
| 6 | Results  More than of half the participants were female. Does this proportion reflect the epidemic of HIV in Yangong? | Among 54,515 PLHV, 24,132 (44.3%) were women with PLHIV in Yangon. This was reported in “Progress Report 2018” by the National AIDS Programme, Ministry of Health and Sports, Myanmar. This discrepancy was inevitable in the study design and explained as a limitation.  “Second, PLHIV were purposively selected by staff at the ART clinics, without involvement by the authors. That is, data on the number of recruited and refused to participate in the study were not available. Also, the number of PLHIV participated at the ART clinics did not reflect the size of the clinics. The majority of PLHIV recruited in this study was from NGO type clinics. These may affect the representativeness of this study.” (line 289-294, page 18) |
| 7 | The majority of patients (82.5%) were recruited from a single ART clinic (NGO). It is likely that the current results reflect the practices of this single ART clinic. The authors should elaborate more on this in the discussion section. | We explained the practices of clinics in the Discussion session.  “Being self-help group members was not associated with having depressive symptoms. However, members who registered at NGO clinics had a lower risk of having depressive symptoms, compared to members who registered at NAP/CBO clinics. The NAP/CBO clinics had fewer human and material resources than NGO funded clinics. Some members who registered at NAP/CBO clinics volunteered in these clinics, for example, for tracing the lost-to-follow-up of PLHIV. This might cause stress and burden on the members. Health professional at NAP/CBO clinics may need an opportunity to learn from NGO clinics regarding how NGOs were supporting self-help groups.” (line 227-235, page 16)  The majority of PLHIV (82,5%) were recruited from NGO running clinics, and this limited the representativeness of this study. We explained this limitation in limitation section as follows:  “Second, PLHIV were purposively selected by staff at the ART clinics, without involvement by the authors. That is, data on the number of recruited and refused to participate in the study were not available. Also, the number of PLHIV participated at the ART clinics did not reflect the size of the clinics. The majority of PLHIV recruited in this study was from NGO type clinics. These may affect the representativeness of this study.” (line 289-294, page 18) |
| 8 | There are a number of differences (education; LGBT; internalized stigma) in the stratified analyses by member status. It is important to control for the variable “type of clinics” to explore its potential role in those documented differences in factors associated with depression my self-help group membership status. | We added the variable “type of clinic” in our analysis. Please see updated result and Table 3. |
| 9 | The second sentence of the discussion “Particularly, those who registered in a government-registered or community-based organization clinic were at higher risk” is not supported by the reported results. | We accepted this comment and removed this sentence from the manuscript. |
| 10 | Second paragraph: The second explanation regarding the self-help group members were more likely to depressive symptoms appears to be far-fetched. Particularly, the sentence “If the members improve their depressive symptoms they could share their experience with the non-members”. Assuming that experience-sharing affect the non-members; it should also affect the members who have joined the self-help group. | Reflecting the change in the model that included the variable “type of clinic,” being members of self-help groups were no longer statistically significant. We removed the part of discussion about this significance. |
| 11 | Conclusion  “Participation in self-help group was not enough to mitigate depressive symptoms although it was found to mitigate internalized stigma”. This sentence implies the notion of causality. This is cross-sectional study; the documented associations cannot infer causality. I suggest the authors to rephrase the sentence and keep to the word “association” and avoid the verb “to mitigate”. | We removed a sentence that implied the causality from the Conclusion section and modified descriptions in the entire Conclusion section. |
| 12 | Minor comments  Any explanation why the being a LGBT was associated with depressive symptoms only among non-members? | We modified the discussion regarding gender and depressive symptoms, including the description of LGBT as follows:  “HIV-infected women and LGBT face stigma and discrimination in their daily lives. Under persistent social norm related to gender, once women and LGBT are infected with HIV, they may perceive a higher level of stress than men. To address their stress, health professional needs to take proactive roles in raising gender equality awareness in the community and encouraging family members of PLHIV to respect gender diversity.” (line 248-253, page 17) |
| 13 | Why did you exclude patients with TB co-infection? | Most (70%) of the interviewers were also PLHIV in this study. PLHIV with TB co-infection was excluded from this study to prevent transmission. |
| 14 | The cross-sectional design should be cited among the limitations of this study. With this design, it is not possible to ascertain causality of the documented associations. | We added more explanation in the limitation section regarding the cross-sectional design of this study.  “First, this study was conducted under a cross-sectional design and it cannot examine exposure and other variables as the causes of depressive symptoms.” (line 286-288, page-18) |
| 15 | Please include line numbers to facilitate review of the manuscript. | We include the line numbers in the amended manuscript. |

**Reviewer #4**

| **No** | **Reviewer’s comment** | **Authors’ response** |
| --- | --- | --- |
| 1 | Major limitation of the study: Being a member, does not translate into or warrant that the person is actually having an active participation. The study did not measure the frequency of attending the self-help group meetings, consistency of participation, or the actual role/activity that the participants are involved in, which may provide a clearer picture of the “sense of belonging” to the self-help group, for us to be able to draw an association between participation and depressive symptoms. There is no measure of “sense of belonging” or level of involvement to the group. Hence, the conclusion (as stated in the of the abstract) that “participation in a self-help group was not enough to mitigate depressive symptoms” might be invalid, as the study did not measure the actual participation but a membership status only. As the authors have stated on page 16 line 4, “The quality of the membership experience may be more important”, yet there was no attempt by the authors to measure the quality of the membership experience rather than measuring the membership status only. Furthermore, the conclusion that highlighted the importance of “additional perceived social support” was too general and does not provide meaningful guideline for public health intervention. It would have been interesting to rather see “where” can we actually intervene; to strengthen the support from medical personnels? or family? or friend? or the self-help group? | This study did not use the collected data on the frequency of attending self-help group meetings and activities. However, the activities of self-help groups were not identical. Some groups have been formed for one decade while others have been for one year. Depending on the relationships with donor agencies, some groups have funding while others have not received anything. Therefore, based on the observations of self-help groups, we judged that the frequency of attending self-help group meetings and the length of membership might not be a proxy for the exposure to the activities or the sense of belongings.  Regarding our implications on enhancing social support, we modified the Conclusion section as follows:  “In this study, self-help groups served as venues where PLHIV interacted with each other and discussed their challenges, including those who had depressive symptoms and a low level of social support.” (line 308-311, page 19)  “As the level of social support was low, particularly among the members of self-help groups, PLHIV should be encouraged to gain more social support through the activities of self-help groups and the services at the ART clinics. These findings should be shared among NAP and NGO clinics to accelerate their activities to mitigate depressive symptoms among PLHIV. Further prospective longitudinal studies are needed to follow up changes in depressive symptoms after participation in a self-help group.” (line 323-329, page 19-20) |
| 2 | Minor comments: Methods: 1.“A two-stage sampling”, may imply stratified and clustered sampling which requires rigorous complex sample analyses. If the authors do not intend to carry out complex sampling analyses, I would suggest that the term is omitted from the manuscript. | We have removed this “A two-stage sampling” from the manuscript. |
| 3 | Has the MOS-SSS been validated in Burmese? | The Burmese version of Medical Outcome Study Social Support Survey (MOS-SSS) was used in our study. This MOS-SSS has already been applied in refugee camps along Thai-Burma Border. Not only Burmese but also Karen versions are available. We inserted the following paragraph in the manuscript.  “The Cronbach’s alpha coefficients for subscales in the Burmese version were 0.87 on the emotional subscale, 0.85 on the tangible subscale, 0.87 on the affectionate subscale, and 0.82 on the positive social interaction subscale.” (line 125-127, page 8) |
| 4 | Was there an incentive to participate in the study? What were the benefits of participating in the study? | To compensate time for the interview, small incentive (soap and towel) was presented the participants.  “PLHIV who participated in this study received soap and towel at the end of the interview.” (line 94-95, page 7) |
| 5 | Participants who are single and separated/widow may have different characteristics and hence, a different prevalence of having depressive symptoms. Not sure why they are grouped together. | We understand that non-marital statuses have different forms, namely, single, separated, and widowed. In a large-scale survey, these could have different categories. However, since the sample size is not large in this study, the number of PLHIV that were fallen into each of the non-marital status categories were small. Consequently, independent categories for single, separated, and widowed might capture the unobserved characteristics of certain individuals had, rather than the non-marital status of single, separated, or widowed themselves. And in a multiple regression analysis, having a category with a few individuals might result in very wide confidence intervals or inability of estimation. Therefore, we decided to integrate these non-marital categories into a single integrated category.  Single, separated, and widowed have common characteristics in a sense that they could not seek for support from their partners. In Myanmar, single, separated, and widowed person tend to live together with sibling or parents and could seek support from these family members. |
| 6 | Age grouped in age-group category rather than age as a continuous variable. Similarly, for an easier interpretation, would it be possible for the authors to categorised other continuous variable such as perceived social support (Low/High), Internalized stigma (Low/High), etc? | We understand the advantage of converting a continuous variable into a dichotomized variable and that health scientists often prefer dichotomization. However, prominent statisticians do not necessarily support the dichotomization due to the loss of rich information that the original continuous variable has.  Please refer to the following article:  Altman DG et al. The cost of dichotomising continuous variables. BMJ. 2006 May 6; 332(7549):1080. |
| 7 | Was there an association between perceived social support and internationalized stigma? Would the authors suggest there is an interaction between having depressive symptoms, perceived social support, and internalized stigma? | Among covariates in the regression model in this study, perceived social support was negatively associated with internalized stigma in bivariate analysis. However, since the interaction term between social support and internalized stigma were not statistically significant, we decided not to incorporate an interaction term between them. |
| 8 | Discussion:  Second paragraph does not at all provide a good argument for the counterintuitive finding; “First, being depressed might cause PLHIV to join self-help groups..” and “Second, the members who had improved….invite depressed peers to join their self-help group”, however, the participants in this study have been in the self-help group for over a year (that was the inclusion criteria), they are not new members to the group. Similarly, page 16 last sentence, these participants have been in the group for over a year. | After including “Type of clinic” variable in the regression analysis according to other reviewers’ comments, being self-help group members was not associated with having depressive symptoms. We rewrote the entire paragraph.  Under the cross-sectional design of this study, we do not have information on the pre-membership levels of depressive symptoms, social support, and internalized stigma. Nevertheless, based on the findings regarding the current levels of these measurements, we tried to list up possible reasons regarding what could happen before their membership, by referring to previous studies. Please kindly note that the list of possible reasons did not guarantee that it really happened in the study site. |
| 9 | Language:  Generally well-written, though improvements can be made. Grammatical errors here and there. Will leave this to the editorial office. | We have checked grammatical errors and corrected them accordingly. |
