# Supplementary material for: Assessing depressive symptoms among people living with HIV in Yangon city, Myanmar: Does being a member of self-help group matter?
Source: PLoS One. 2021 Mar 18;16(3):e0248807. doi: 10.1371/journal.pone.0248807 (PMC7971502; doi:10.1371/journal.pone.0248807)
Supplement: Attachment — Submitted filename: Responses to Reviewers_Final.rtf [file pone.0248807.s003.rtf]

Responses to reviewers

Thank you very much for taking the time to review the manuscript. We responded to your valuable comments as below:

No	Reviewer's comment	Authors' response	
1.	Abstract
As recommended by UNAIDS, people (such as PLHIV) should never be
referred to as an abbreviation since this is dehumanizing. Instead,
the name or identity of the group should be written out in full.
Please see:
https://www.unaids.org/sites/default/files/
media_asset/2015_terminology_guidelines
_en.pdf 	We amended the manuscript by using the term, people living with HIV, not PLHIV. 	
2.	'Data' is plural form of 'datum.' Please use a plural verb with it;
e.g., data were collected…	We corrected the usage of the term, data, as suggested in the entire manuscript. 	
3.	Methods: '…as well as other factors' may be unnecessary. Instead,
please keep the space to briefly describe sampling methods.
Please double-check 95% CI in this sentence: The membership was not
associated with having depressive symptoms (adjusted odds ratio [AOR]
1.59, 95% confidence interval [CI] 1.98 - 2.59), which appeared
incorrect.	We removed 'as well as other factors' and added the description of sampling methods in the Abstract. 
“Three ART clinics were purposively selected based on the recommendation from the National AIDS Program in Myanmar. At these clinics, people living with HIV were recruited by a convenience sampling method.” (lines 29-31, page 1)

We check 95% CI and corrected accordingly. (lines 33-35, page 1) 
“The membership was not associated with having depressive symptoms (adjusted odds ratio [AOR] 1.59, 95% confidence interval [CI] 0.98 - 2.59).”	
4.	Conclusions: To save space for other essential information, the
conclusions can be more condensed. For instance, the first and second
sentences are too broad and do not deserve space.
Please remove the keywords. We need them only in the submission
system. Also, 'people living with HIV' should not be capitalized.
	We rewrote the first and second sentences as follow,
“This study did not find evidence on the association between membership in self-help groups and depressive symptoms among people living with HIV. Social support was a protective factor against depressive symptoms both self-help group members and non-members, although the level of social support was lower among members than non-members. The activities of self-help groups and care provided by the ART clinics should be strengthened to address mental health problems among people living with HIV in the study site.” (lines 40-46, page 2)

We have removed keywords from the Abstract. 	
5.	Introduction

First sentence: Not sure 'chronically manageable' is what the authors
intended to say. Consider saying, 'HIV infection has become a chronic,
manageable condition.'
	We amended the sentence as suggested.
“As antiretroviral therapy (ART) access has been improved, HIV infection has become chronic, manageable condition.” (lines 75-76, page 3)	
6.	Line 6: Please remove 'In practice.'
	We amended the sentence as suggested.
“Depression is a common mental health disorder among people living with HIV.” (lines 79-80, page 3)	
7.	Lines 13-14: Please remove “In a self-help group of PLHIV,” as it is
redundant and unnecessary.
	We amended the sentence as suggested.
“They encourage each other to talk about their challenges, for example, stigma and discrimination, and advocate measures to address such challenges in the community.” (lines 86-88, page 3)”	
8.	Line 19: Add ',' before 'possibly.'
	We amended the sentence as suggested.
“However, some people living with HIV avoid joining a self-help group, possibly because they want to hide their HIV-infection status.” (lines 92-93, page 3)	
9.	Line 21: Please remove 'In addition to self-help groups' as it does
not go along well in the following statement on social support. Self-help groups are a form of social support.
	We amended the sentence as suggested.
“Social support can play a vital role for people living with HIV in coping with depression.” (lines 94-95, page 3)	
10.	Line 26: 'sub-classified.'
	We amended the sentence as suggested.
“…it is sub-classified into actual support and perceived support.” (line 99, page 4)	
11.	Lines 26-28: The sentence “As the effect of perceived social support
may differ from actual social support, perceived social support is
more important in mental health” is not clear. It's difficult to
understand how the assumption that 'perceived social support' is more
important in mental health is explained by the different effects between actual and perceived social support.	We amended the sentences to clarify differences in the measurement of actual and perceived social support as follows:
“Actual support addresses how family, friends, and co-workers intend to support people living with HIV. However, social support can be effective in coping with depression among people living with HIV once they perceive the support. Therefore, perceived social support may be a factor more directly related to depressive symptoms that people living with HIV have.” (Lines 100-104, page 4)	
12.	Lines 29-30: Suggest revising, “In 2018, the estimated HIV prevalence
among adults aged 15-49 in Myanmar was 0.8%.
	We amended the sentence as follows.
“In 2018, the estimated HIV prevalence was 0.8% among those who aged between 15 and 49 in Myanmar.” (lines 105-106, page 4)	
13.	Lines 30-31: Please consider revising the sentence: “Of the total
240000 people living with HIV in Myanmar, xx% received ART in xxxx.”
	We added information as follows:
“Of the total 240,000 people living with HIV in Myanmar, 70.8% received ART in 2018.” (lines 107-108, page 4)	
14.	Lines 32-33: Please add ',' before 'and.' Also, this information is
not clear, “Despite these improvements in care, 60.0% of PLHIV feel
ashamed and 18.0% are denied access to sexual and reproductive health
services due to their HIV status in Myanmar.” What do people living
with HIV feel ashamed of? By whom are they denied access to SRH
services?
	We amended the sentence as suggested: 
“Despite these improvements in care, 60.0% of PLHIV feel ashamed of being infected with HIV infection and 18.0% are denied access to sexual and reproductive health services by health care providers due to HIV status in Myanmar.” (lines 108-111, page 4)	
15.	Line 37: Please double check the measurement of depression. Was it
clinical depression or depressive symptoms? 30% prevalence of
depression seems too high. Also, when mentioning any 'prevalence,'
please specify the population – was it a national prevalence?
	We amended the sentence as necessary. In that study, Center for Epidemiology Studies Depression Scale (CES-D) was used to measure depressive symptoms among (425) people living with HIV in Yangon, Myanmar. It was measuring depressive symptoms and it was not national prevalence data. And we amended as follow,
“The prevalence of depressive symptoms among people living with HIV was as high as 30.1% in one study conducted Yangon City, Myanmar” (lines 114-115, page 4)	
16.	Line 40: Please remove or specify 'To clarify that' – what is 'that?'
	We amended the sentence as suggested: 
“Studies are needed to address the level of supports that help people living with HIV to cope with depression.” (lines 118-119, page 4)	
17.	Methods


Line 59: …in Yangon, Myanmar, in 2017.
Line 60: … from August to September 2017.	We amended the sentences as suggested: 
“A comparative cross-sectional study was conducted in Yangon city, Myanmar, in 2017. The data were collected from August to September 2017.” (lines 137-138, page 4)	
18.	Lines 60-61: Please revised this sentence: “Yangon was selected as the
study site because this city reported many HIV cases.” Instead of
saying “many HIV cases,” please provide the actual number of total
cases or the proportion of people living with HIV in Yangon relative
to the national people living with HIV population size.
Please be consistent and specific: Is Yangon different from Yangon city?
	We revised the justification for selecting Yangon city as the study site as suggested, although the sub-national statistics were scarce in Myanmar. 

“Yangon city was selected as the study site since the city was expected to have a large number of key populations, particularly vulnerable with HIV and those who received ART, although sub-national statistics related to HIV are scarce in Myanmar. A total of 23,347 people living with HIV joined self-help groups in Myanmar, and 6,339 of them were members of self-help groups in Yangon city in 2015. As approximately 40% of total people living with HIV on ART were living in Yangon region in 2015, several international non-governmental organizations (NGOs) and ten government health teams implement HIV-related services in Yangon city.” (lines 138-146, pages 5-6)	
19.	Line 65: What did the authors refer to by 'public teams?'
	In Myanmar, 'public teams' mean 'government health teams'. We have amended the words as suggested. 
“….non-governmental organizations (NGOs) and ten government health teams implement….” (line 145, page 6) 	
20.	Line 67: “This study was conducted in ART clinics.” Please also
indicate the number of ART clinics in the city and the study, and how
they were selected.
	In Myanmar, 127 ART clinics were providing health care service to people living with HIV (reference: Myanmar Progress report, 2018). We tried to figure out the number of ART clinics in Yangon city. However, there was no official list published by National AIDS Program in Myanmar. We clarified the selection process as follows:
“In this study, recruitment process was as follows: First, three ART clinics were purposively selected through consultation with the National AIDS Program (NAP). Assistant Director from NAP recommended three ART clinics that cooperated well with NAP and sent a regular report to NAP.” (line 165-168, page 6-7) 	
21.	Lines 67-69: The sentence “A structured questionnaire was administered to all of the participants through face-to-face interviews in Burmese
language by trained interviewers in private rooms at the ART clinics”
should not be placed under the 'Study setting.” Also, it was not clear
whether 'all participants' refer to a 'take-all” approach. If not, a
sampling procedure should be described.	We moved the sentence from “Study setting” to “Participants, recruitment, and data collection”. 

“A structured questionnaire was administered through face-to-face interviews in Burmese language by trained interviewers in private rooms at the ART clinics.” (lines 174-176, page 7)

Sampling procedure was explained as follows:

“Second, people living with HIV were recruited by a convenience sampling method when they visited the clinics during the data collection period. The clinic reception staff announced this study in the waiting area. Those who were interested in the participation received explanations from the staff on the purpose of this study. Only those who were willing to participate in this study were sent to the interview rooms. People living with HIV who participated in this study received soap and towel at the end of the interview.” (lines 168-174, page 7)
	
22.	Line 71: … ; the clinic provided ART and counseling. Also, please
specify the 'counseling' – HIV confidential counseling and testing or
other counseling services?
	ART clinics provide counseling, such as pre-test counseling, post-test counseling, and ART adherence counseling. We clarified counseling activities in ART clinics as follows:
“When people were diagnosed with HIV positive, the clinic provided ART and counseling, such as pre-test, post-test and ART adherence counseling.” (lines 149-151, page 6) 	
23.	Line 80: … and 12.8 for the standard care group. The calculation
provided a required sample size of…	We amended the sentence as follows:
“…..and 12.8 for standard care group. The calculation provided a required sample size of….” (lines 158-159, page 6)	
24.	Lines 81-82: It appeared that the inclusion criteria are not exhaustive. The wording for inclusion and exclusion criteria should be improved. Also, please provide the reason why people with HIV/tuberculosis co-infection were excluded.	We extensively modified the sentences for inclusion and exclusion criteria as follows:
“This study included people living with HIV aged 18 years or above who were enrolled in ART clinics at Yangon city. Among them, this study excluded those who were co-infected with tuberculosis for the purpose of preventing further transmission of infections to interviewers. It also excluded critically ill people living with HIV who could not answer the questionnaire. ” (Lines 160-164, page 6)

In this study, four interviewers among total eight interviewers were people living with HIV. We would like to sure prevention of tuberculosis transmission to our interviewers. This was the main reason to exclude people with HIV/tuberculosis co-infection.	
25.	Line 86: In this study, the recruitment process is as follows…
The first step in the participant recruitment process may introduce
serious selection bias that affects the study population's representativeness. I would suggest highlighting it in the
limitations.
	We added the following sentence under the “Limitation” section.
“ Second, ART clinics and people living with HIV were purposively selected by NAP and staff at the ART clinics without involvement by the authors.” (lines 381-383, page 20) 	
26.	Lines 89-90: Please elaborate on how the 'convenience sampling method'
was performed.
	The responsible persons at the ART clinics did not allow us to recruit directly to people living with HIV enrolled at the clinics. They protected their patients' identity and strongly kept confidentiality. They announced the study at the waiting area and those who were interested to participate in our study, they sent these persons to the interview rooms. We modified the explanations as follows:

“Second, people living with HIV were recruited by a convenience sampling method when they visited the clinics during the data collection period. The clinic reception staff announced this study in the waiting area. Those who were interested in the participation received explanations from the staff on the purpose of this study. Only those who were willing to participate in this study were sent to the interview rooms. People living with HIV who participated in this study received soap and towel at the end of the interview.” (lines 168-174, page 7)	
27.	Line 97: Please remove “To assess the membership status of self-help
group [41],” which is unnecessary and somewhat redundant.
Line 98: … being a member of a self-help group.	We removed and modified the sentences as suggested:
“This study measured 1) being a member of a self-help group and 2) the length of their membership in years” (lines 178-179, page 7)	
28.	
Line 99: Please remove 'presence of.'
Line 103: Please replace ';' by '.'
	We amended the sentences as you suggested. 
“This study used the Hopkins Symptom Checklist for Depression (HSCL-D) to measure the severity of depressive symptoms” (lines 182-183, page 7)

“The Burmese version of HSCL-D (Cronbach's alpha: 0.85) has already been validated among Myanmar refugees along the Thai-Myanmar border.” (lines 183-185, page 7)	
29.	Line 105: An answer to each question was given…
Lines 107-108: The minimum mean score was one, and the maximum mean
score was four.
	We amended the following sentences as suggested:
“An answer to each question was given on a four-point Likert-type scale (1=not at all, 2=a little, 3=quite a bit, 4=extremely).” (lines 187-188, page 8)
“The minimum mean score was 1, and the maximum mean score was 4.” (line 188-190, page 7)	
30.	Line 116: How was ART adherence measured?
	At ART clinics, we did not have a chance to review the treatment record of people living with HIV due to confidential issue. Therefore, we collected ART adherence through face-to-face interview questions.

We added the explanations as follows:
“ART status was measured based on the interview questions on the regular intake or not, the frequency of intake, and the number of tablets per intake. ART adherence was measured based on an interview question on any missing of intake in the previous week of data collection.” (lines 200-203, page 8)	
31.	Line 119: …, with a total score ranging from 0 to 7.
	We amended the sentence as follows:
“Each item was rated dichotomously (1=agree or 0=disagree); with a total score ranging from 0 to 7.” (lines 204-205, page 8)	
32.	Line 123: Please specify the value in the brackets (0.91). Is it a
Cronbach's alpha?
	We amended the sentence to clarify that Cronbach's alpha was mentioned as follows:
“…..its Cronbach's alpha was 0.91” (lines 208-209, pages 8-9)	
33.	Line 133: 'Chi-square test' should be capitalized.
	We amended the sentence as suggested:
“A Chi-square test or Fisher's exact test…” (line 219, page 9)	
34.	Line 137: Bivariate analyses were conducted… Also, please specify what
the bivariate analyses are – are they different from the bivariate 
analyses mentioned above?
	In addition to t-test, Chi-square test, and Fisher's exact test, we conducted bivariate logistic regression analyses to select covariates to be included in the multiple logistic regression analysis. We amended the explanations as follows:
“ Multiple logistic regression analysis was conducted to find factors associated with having depressive symptoms. As the covariates of the regression model, only statistically significant variables at 5% level found in bivariate logistic regression analyses were included among variables for HIV-related characteristics, perceived social support, and the type of a clinic. In addition, all of the socio-demographic factor variables mentioned in the previous sub-section were included as covariates.” (lines 223-228, page 9)	
35.	Line 138: Only the variables associated with…
	We modified the explanation as follows:
“As the covariates of the regression model, only statistically significant variables at 5% level found in bivariate logistic regression analyses were included among variables for HIV-related characteristics, perceived social support, and the type of a clinic.” (lines 224-227, page 9)	
36.	Lines 138-140: The selection of the covariates for multiple regression
analyses was not clear. What was the cut-off to define the
significance level in bivariate analyses? Were all socio-demographic
factors included in the models?
	The cut-off was p-value 0.05 to define the significance level in bivariate analyses. All socio-demographic factors were included in the models. To clarify these points, we modified the explanations as below:
“As the covariates of the regression model, only statistically significant variables at 5% level found in bivariate logistic regression analyses were included among variables for HIV-related characteristics, perceived social support, and the type of a clinic. In addition, all of the socio-demographic factor variables mentioned in the previous sub-section were included as covariates.” (lines 224-228, page 9)	
37.	Lines 147-149: Please provide a reference number for ethics approvals.
	We added the information as suggested:
“This study was approved by both the Research Ethics Committee of the Graduate School of Medicine at the University of Tokyo (11647) and the Ethics Review Committee of the Department of Medical Research in Myanmar (Ethics/DMR/2017/111)” (lines 235-238, page 10)	
38.	Line 153-154: Confidentiality was maintained by not recording any
personal identifiers in....
	We modified the sentence as follows:
“Confidentiality was maintained by not recording any personal identifiers in all instruments and analyses.” (lines 242-243, page 10)	
39.	Results

Line 157: Please revise 'PLHIV characteristics' to 'Participant
characteristics.'	We amended the title of the sub-section to “Participant characteristics.” (line 246, page 11)	
40.	For dichotomous variables, presenting only one group is sufficient.
Line 162: …, and of non-members was…
Line 169: …, which was lower…
Line 170: Remove ',' before 'and 65.1'	We modified the sentences as follows:
“The mean age was 39.2 (SD 9.5) years among members and 38.7 (SD 9.6) years among non-members.” (lines 252-253, page 11)
“The mean score of internalized stigma was 0.6 (SD 1.1) among members, which was lower than the mean score of non-members, 1.0 (SD 1.5).” (line 258-259, page 11)
“The mean perceived social support score was 53.4 (SD 29.5) among members and 65.1 (SD 32.1) among non-members.” (line 259-261, page 11)
	
41.	Table 1: Self-help group members, non-members
Line 194: The length of more than one year in a self-help group…
Lines 194-195: Non-significant results may not be presented.
Table 3: Self-help group members, non-members
	We corrected the column headings as suggested in Tables 1 and 3. 
And we removed the description of non-significant results presented in Table 3 from the main text.	
42.	Discussion


Line 222: However, self-help group members registered at NGO clinics…
Line 223: … symptoms compared to members at…


	We modified the sentences as suggested:
“However, self-help group members registered at NGO clinics had a lower risk of having depressive symptoms compared to members at NAP/CBO clinics” (lines 311-313, page 17)	
43.	Line 228: However, members registered at…
Line 229: … of having depressive symptoms compared to…
	We modified the sentence as suggested:
“However, members registered at NGO clinics had a lower risk of having depressive symptoms compared to members who registered at NAP/CBO clinics.” (line 318-319, page 17)	
44.	Line 240: … and what duration they want…


	We modified the sentence as suggested:
“…and what duration they want to participate in the …..” (line 330, page 17)	
45.	Line 245: In this study, gender differences existed in…
Line 246: …non-members, consistent with previous studies
Line 249: Under persistent social norms related…	We modified the sentence as suggested:
“In this study, gender differences existed in depressive symptoms among members and non-members, consistent with previous studies” (line 335-336, page 18)
“Under persistent social norms related to gender….” (line 339, page 18)	
46.	Line 254: Please be consistent in the terminology use – depression or
depressive symptoms
	We modified the sentences as suggested:
“Lower education level was associated with having depressive symptoms among members. This association was not found among non-members. The effect of participating in self-help groups on depressive symptoms might be affected by members' education level.” (line 344-347, page 18)	
47.	Line 262: … symptoms, both among the members and non-members	We modified the sentence as suggested:
“…symptoms, both among members and non-members” (line 353, page 18)	
48.	Line 277: Please use consistent English – British or American; e.g., counseling
	We reviewed the manuscript to ensure that “counseling” was consistently used.
	
49.	The writing quality of the limitations section needs improvement as it
is the most difficult to read.
Line 287: …, and it cannot…
Line 292: Also, the number of PLHIV who participated at
Line 299: …The investigation of reasons…	We amended the entire limitation section to improve readability and clarity.
	
50.	The whole conclusions section requires improvement. The first few
sentences are too broad and should summarize key findings. The third
and fourth sentences are the same. Please avoid repeating results and
discussions in this section.
	We amended the entire Conclusion section as advised to highlight the findings and avoid duplications in the explanations. 
	
51.	References

The references need improvement as they are not consistent and not
aligned with PLOS' guidelines.	We have amended the references according to PLOS' guidelines.
